# A monocyte/granulocyte to lymphocyte ratio predicts survival in patients with hepatocellular carcinoma

**DOI:** 10.1038/srep15263

**Published:** 2015-10-21

**Authors:** Dongsheng Zhou, Yaojun Zhang, Li Xu, Zhongguo Zhou, Junting Huang, Minshan Chen

**Affiliations:** 1 Department of Hepatobiliary Surgery, Sun Yat-sen University Cancer Center, 651 Dongfeng Road East, Guangzhou 510060, China; 2 Sun Yat-sen University Cancer Center, State Key Laboratory of Oncology in South China, Collaborative Innovation Center for Cancer Medicine, Guangzhou 510060, China

## Abstract

Conflict that the derived neutrophil lymphocyte (dNLR) has prognostic value in patients with a variety of cancers exists. The aim of the present study was to devise a monocyte/granulocyte to lymphocyte ratio (M/GLR) which counts as (white cell count - lymphocyte count) to lymphocyte count, and verify its prognostic value in patients with hepatocellular carcinoma (HCC). 1061 HCC patients were retrieved and the associations between M/GLR/NLR/dNLR and clinicopathological variables and survivals (OS and RFS) were analyzed. The area under the curve (AUC) was calculated to evaluate the discriminatory ability of M/GLR/NLR/dNLR. The median follow-up period was 947 days, the 1, 3, 5 year OS was 64%, 51%, and 46% respectively, and the median OS was 842 days. The cut-off values were determined by ROC as 2.8, 1.6, and 3.2 for NLR, dNLR, M/GLR respectively. Elevated M/GLR/NLR/dNLR was associated with poor prognosis (P = 0.001, P = 0.009 and P = 0.022 respectively). By time-dependent ROC, the AUC of M/GLR was higher than that of NLR or dNLR, either in whole group or in subgroups according to TNM stages or different treatments. We concluded that elevated M/GLR predicted poor prognosis for patients with HCC and the M/GLR can be used as an alternative to NLR and dNLR.

Hepatocellular carcinoma (HCC) is the fifth most common neoplasm and the second leading cause of cancer-related deaths worldwide, with the incidence rising in the world^1^,^2^,^3^. Besides genetic basis, environment factors play an important role in neoplastic process. As the last and most redoubtable clinical consequence of cirrhosis, most HCC is related to chronic viral infection. Recent studies have shown that host inflammatory response plays an important role in carcinogenesis and disease progression^4^,^5^.

With growing evidences on the role of inflammation in cancer biology, a systemic inflammatory response has been revealed as having prognostic significance in a variety of cancers. NLR, GPS, mGPS, PLR, PNI and PI have been shown to have prognostic value in patients with a wide range of cancer types^6^,^7^,^8^,^9^,^10^,^11^,^12^,^13^,^14^,^15^,^16^,^17^,^18^. NLR, from neutrophil and lymphocyte counts, can be easily obtained from day-to-day oncological practice without expensive measurement costs. These two components made it more practical, although inferior to other systemic inflammatory scores. Clarke *et al.*^19^ indicated that if those extensive data were to confirm the prognostic value and clinical utility of the NLR, it would be an important, relevant, clinical translational advance in the identification of cancer patients at high risk.

Recently, to resolve lack of databases in multicenter randomized controlled clinical trials, Proctor *et al.*^20^ hypothesized a derived neutrophil to lymphocyte ratio (dNLR) counted as neutrophil count to (white cell count –neutrophil count), which is composed of only a white cell and neutrophil count. In that study, 12118 patients with different kinds of cancers were recruited. They evaluated the prognostic value of dNLR on overall survival and cancer-specific survival, and demonstrated that the dNLR had similar prognostic value with NLR. Recently, external validation of this prognostic risk assessment tool in independent cohorts was performed. But following conclusions were inconsistent. Studies confirmed elevated pre-treatment dNLR as an independent prognostic factor in patients with pancreatic cancer^21^ and diffuse large B-cell lymphoma^22^. However, others suggested that dNLR was not independently associated with survival in patients with breast cancer^23^ and gastric cancer^24^. Besides neutrophil and lymphocyte, monocyte is another part mainly consisting of leucocyte. Studies have shown that monocyte is an independent prognosis factor, and higher monocyte predicts poor prognosis, which has the same effects as neutrophil in predicting prognosis. But dNLR puts the WBC-neutrophil, mainly consisting of lymphocyte and monocyte with possible opposing effects in terms of predictive value, as denominator. It may be the reason why we can’t get the consistent results in variation studies. Here, we postulated a monocyte/granulocyte to lymphocyte ratio (M/GLR), which counts as (white cell count - lymphocyte count) to lymphocyte count, using neutrophil and monocyte together as numerator. The purpose of this study was to evaluate the efficiency, feasibility and prognostic value of M/GLR for patients with HCC, and compare to those of NLR and dNLR.

## Material and Methods

### Patients

Patients who were hospitalized and treated with surgical resection, ablative therapy and transcatheter arterial chemoembolization (TACE) for HCC from 2007 to 2009 at the Department of Hepatobiliary Surgery, Sun Yat-Sen University Cancer Canter (Guangzhou, China) were identified from our prospective database. The research was approved by the institutional review board (IRB) of Sun Yat-sen University Cancer Center. At the time of submission, written informed and consent were obtained from the participants or their parent or legal guardian. All treatments were performed in accordance with relevant guidelines and regulations. The diagnosis of HCC was based on the diagnostic criteria for HCC used by the American Association for the Study of the Liver (AASLD) guideline^25^. HCC was diagnosed by at least two radiologic images showing characteristic features of HCC or one radiologic image showing characteristic features of HCC associated with elevated serum AFP (≥400 ng/mL) or histopathologic evidence.

Patients who met all of the following criteria were included in this study: 1) clinical and histologic confirmation of HCC; 2) no previous treatment before surgery, ablation, or TACE; 3) Liver function was Child-Pugh A or B; 4) follow-up ≥3 months. 5) no inflammation or diseases causing high leukocyte before treatments; Patients with other cancers were excluded from this study.

All the parameters were recorded and evaluated as possible predictors of survival including gender, age, white blood cell count (WBC), neutrophil count, lymphocyte count, platelet count (PLT), alpha-fetoprotein (AFP), alkaline phosphatase (ALP), total bilirubin level (TBIL), albumin (ALB), tumor size and number, and tumor thrombus.

### Follow-up

Patients were followed carefully after treatment. Patients underwent liver computed tomography (CT) scans one month after treatment, and liver CT scans were performed at three-month intervals during the first 2 years, then 6 month intervals thereafter with physical examination, blood tests for AFP and liver function. When metastasis was suspected, chest CT, bone scintigraphy, positron emission tomography (PET), and biopsy if indicated were also performed to confirm metastasis. The end of follow-up was the time of last follow-up (January 2014) or death.

### Statistical Analyses

The parameters were constructed as follows: NLR = neutrophil count to lymphocyte count, dNLR = neutrophil count to (white cell count - neutrophil count), M/GLR = (white cell count– lymphocyte count) to lymphocyte count.

The overall survival (OS) was calculated from the time of first treatment to death. The recurrence free survival (RFS) was calculated from the time of first treatment to the first recurrence. Receiver operating characteristic (ROC) curve was used to determine the cut-off value of NLR, dNLR, and M/GLR. The OS and RFS was calculated by Kaplan-Meier method and compared by log-rank test. The prognostic values in predicting OS were assessed by multivariate Cox proportional hazards regression analysis. All covariates that affected survival at the P < 0.10 level of significance in univariate analysis were included in multivariate Cox proportional hazards model. Time-dependent ROC analysis and the area under ROC curve (AUC) were used to compare the capability of the prognostic value among NLR, dNLR, and M/GLR. Spearman rank correlation analysis was used to determine the relationships among NLR, dNLR and M/GLR. Results were given as mean ± S.D. All statistical tests were two-sided, and a significant difference was considered when p < 0.05. All the statistical analysis was performed using the SPSS 19.0 statistical software (SPSS Company, Chicago, Illinois, USA).

## Results

### Baseline characteristics

A total of 1061 patients who met our criteria were included in the present study. Patients’ baseline characteristics were summarized in Table 1. There were 952 male (89.7%) and 109 female (10.3%) patients with a median age of 52 years (range, 13–80 years). The majority of our patients had a good liver functional reserve with Child-Pugh A (96%).

By using ROC analysis, we determined a cut-off value of 2.8 for NLR, 1.6 for dNLR and 3.2 for M/GLR to be the best to differentiate between patients’ survival in whole cohort. According to these cut-off values, 273 patients had NLR > 2.8 (25.7%), 385 patients had dNLR > 1.6 (36.3%), and 296 patients had M/GLR > 3.2 (27.5%) respectively.

### Survival and Prognostic factors

**Figure 1:**
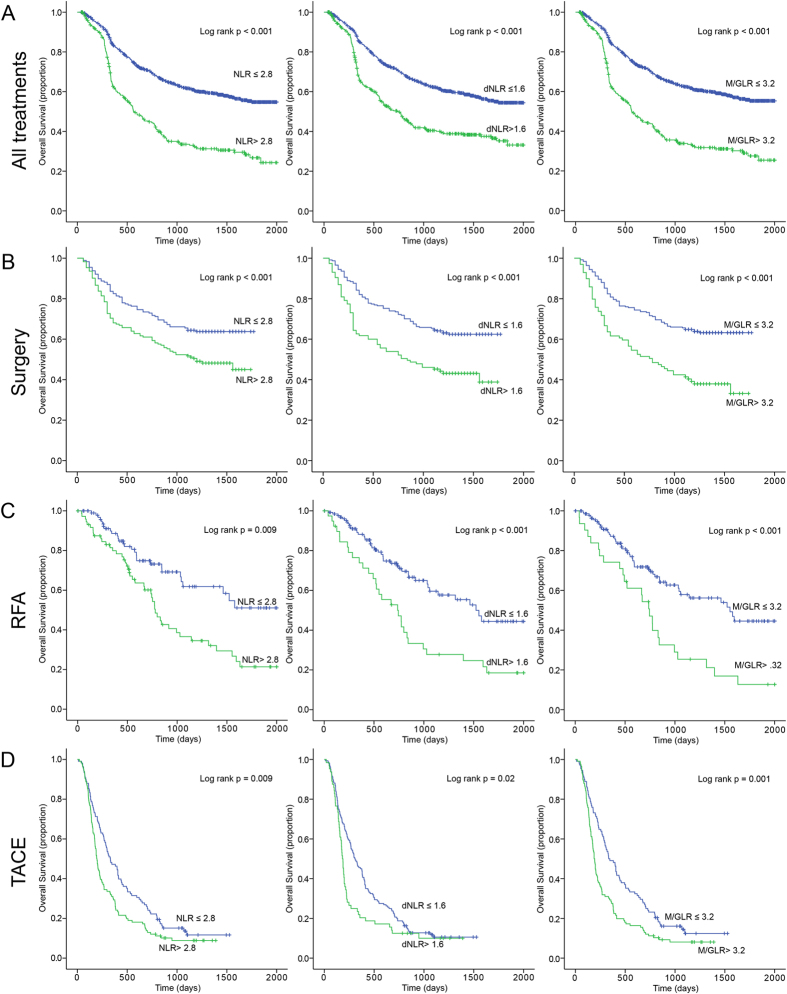
Kaplan-Meier survival curves for overall survival in 1061 patients with hepatocellular carcinoma after treatments. (**A**) All Treatments, (**B**) Surgery, (**C**) RFA, (**D**) TACE.

**Figure 2:**
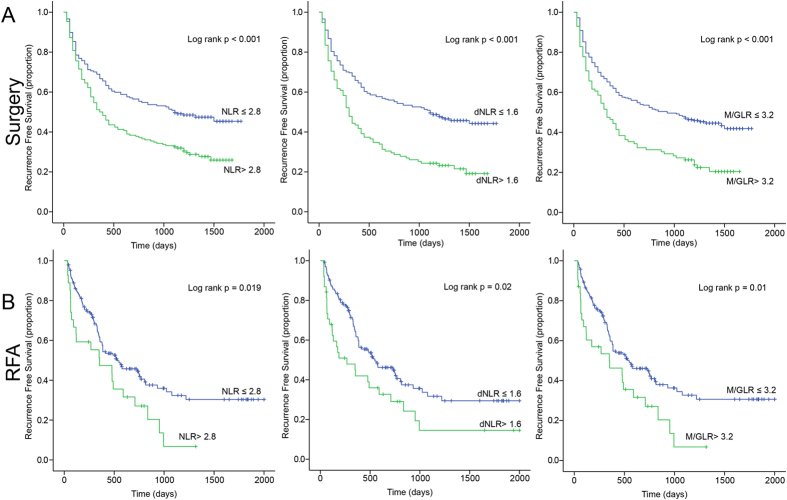
Kaplan-Meier survival curves for recurrence free survival in surgery (**A**) and RFA (**B**).

The median follow-up period was 947 days. The 1, 3, 5 year OS was 64%, 51%, and 46% respectively, and the median OS was 842 days for the whole group. The univariate and multivariate analyses of prognostic factors for OS were analyzed. In univariate analysis (Table 1), WBC (P = 0.001), Neutrophil count (P < 0.001), Lymphocyte count (P < 0.001), ALT (P < 0.001), AST (P < 0.001), ALB (P < 0.001), ALP (P < 0.001), GGT (P < 0.001), TBIL (P = 0.001), AFP (P < 0.001), PT (P = 0.001), PT (P = 0.027), ascites (P < 0.001), tumor size (P < 0.001), tumor number (P = 0.041), vascular invasion (P < 0.001), Child-Pugh scores (p = 0.005), TNM (P < 0.001), NLR (P = 0.001), dNLR (P = 0.001), and M/GLR (P = 0.001). The Kaplan-Meier analysis and log-rank test showed that all three factors were significant in OS (All treatment: NLR P < 0.001, dNLR P < 0.001, M/GLR P < 0.001; Surgery: NLR P < 0.001, dNLR P < 0.001, M/GLR P < 0.001; RFA: NLR P = 0.009, dNLR P < 0.001, M/GLR P < 0.001; TACE: NLR P = 0.009, dNLR P = 0.02, M/GLR P = 0.001, respectively) (Fig. 1). At the same time all three factors were revealed to be significant in RFS after surgery (NLR P < 0.001, dNLR P < 0.001, M/GLR P < 0.001, respectively) and RFA (NLR P = 0.019, dNLR P = 0.02, M/GLR P = 0.01, respectively) respectively (Fig. 2).

Multivariate analysis (Table 2) showed that AFP, diameter of largest lesion, tumor number and vascular invasion were independent prognostic factors for OS, along with NLR (HR 1.32, 95% CI 1.07–1.62, P = 0.009), or dNLR (HR 1.26 95% CI 1.03–1.52, P = 0.022), or M/GLR (HR 1.40 95%CI 1.14–1.71, P = 0.001).

### The associations and comparisons among NLR, dNLR and M/GLR

The associations among NLR, dNLR and M/GLR were assessed by Spearman’s rank correlation analysis. There were significant correlations among three scores (NLR vs. dNLR R = 0.961, P < 0.001, NLR vs. M/GLR R = 0.986, P < 0.001, dNLR vs. M/GLR R = 0.906, P < 0.001). Besides neutrophil, M/GLR contains monocyte and other granulocyte, so M/GLR is consistently higher than NLR.

**Figure 3:**
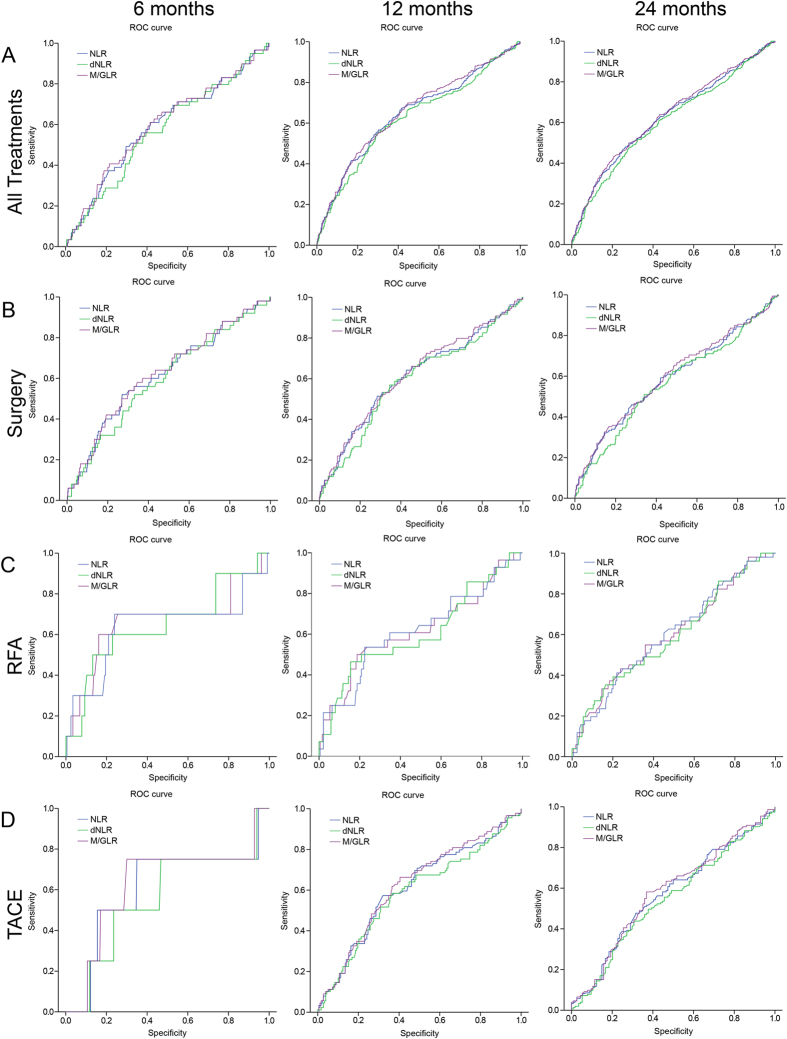
Comparison of the AUC for outcome prediction among NLR, dNLR, and M/GLR for patients with HCC after treatment. (**A**) All Treatments, (**B**) surgery, (**C**) RFA, (**D**) TACE.

**Figure 4:**
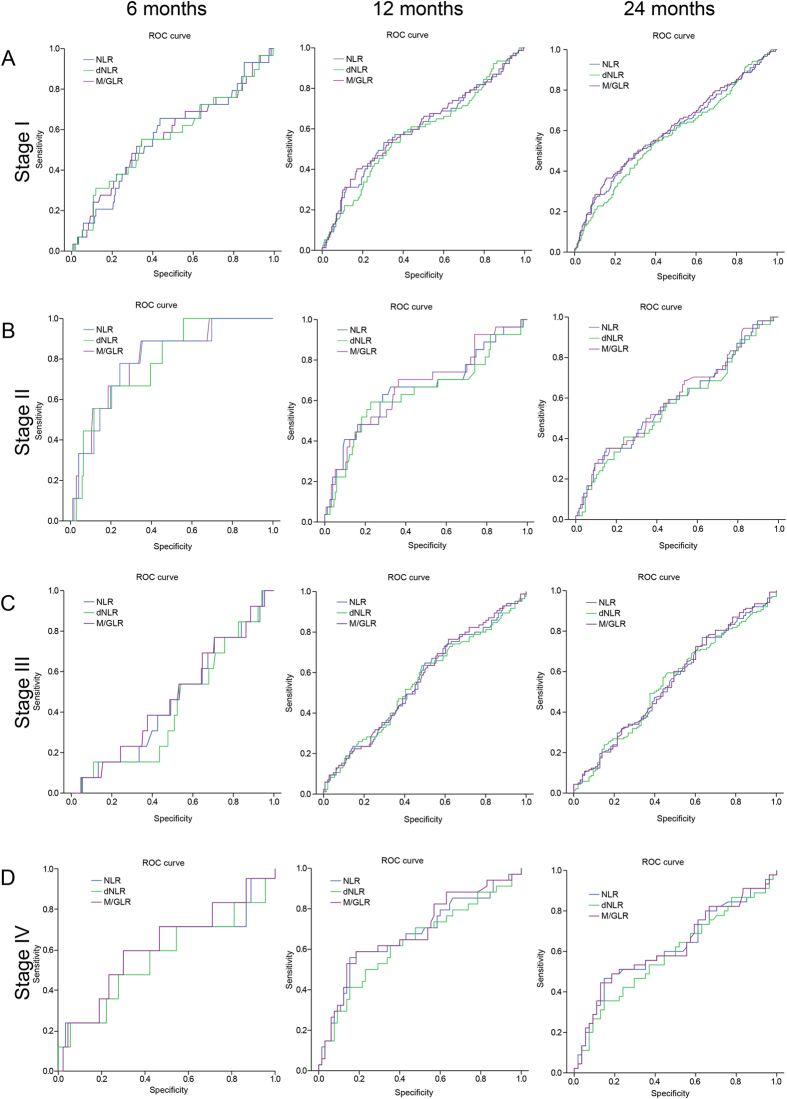
Comparison of the AUC for outcome prediction among NLR, dNLR, and M/GLR for patients with different TNM stages. (**A**) TNM-I, (**B**) TNM-II, (**C**) TNM-III, (**D**) TNM-IV.

Time-dependent ROC analysis and means of AUC analysis was used to compare the prognostic power of NLR, dNLR and M/GLR. As it was shown in Figs 3 and 4 and Supplement Tables 1 and 2, the AUC of M/GLR was higher than that of NLR or dNLR, either in whole group or in subgroups according to TNM stages or different treatments (surgery, ablation and TACE).

## Discussion

In the present study, we postulated a modified NLR (M/GLR), constructed as (white cell count-lymphocyte count) to lymphocyte, and evaluated the prognostic power of M/GLR, NLR and dNLR for patients with HCC. Our results demonstrated that higher M/GLR in untreated patients predicted poor prognosis. AUC analysis and multivariate analysis revealed that M/GLR was superior to NLR and dNLR in prognostic power for patients with HCC, in either whole group or subgroups according to TNM stages or different treatments. Thus the M/GLR can be used as an alternative to NLR and dNLR.

The majority of HCC occurs in patients with chronic liver disease including hepatitis B viral (HBV) infection, chronic hepatitis C virus (HCV) infection, hereditary hemochromatosis, and cirrhosis of almost any cause^26^. Evidences revealed that the inflammatory factors play a crucial role in promoting proliferation, invasion and metastasis of malignant cells^27^,^28^. Different parts of infiltrating inflammation cells in tumor microenvironment play different roles. Neutrophils have been associated with poorer prognosis in many cancers. Atzpodien *et al.*^29^ assessed a total of 495 patients, and concluded that the patients with higher neutrophil counts had shorter median survival (P < 0.001). Neutrophils in peripheral blood or in the tumor microenvironment were shown to produce pro-angiogenic factors including vascular endothelial growth factor to stimulate tumor development and progression^30^. Lymphocytes operate as crucial components of the adaptive immune system and are the cellular basis of cancer immunosurveillance and immunoediting and low lymphocyte counts may be responsible for an inadequate immunologic reaction to the tumor, revealing a weakened defense against cancer with poor prognosis^31^,^32^. Besides the neutrophils and lymphocytes, the monocyte is the third main integral part of leucocyte. It was also reported that a higher monocyte count is associated with poor prognosis in various cancers^33^,^34^,^35^,^36^. Sasaki *et al.*^37^ retrospectively examined the relation between the preoperative absolute number of peripheral monocytes and long-term prognosis in 198 patients with operable HCC. They showed that higher peripheral monocyte count was an independent risk factor and indicated worse 5-year disease-free survival rate.

NLR had been revealed as an independent prognostic factor for patients with different kinds of cancers, with the advantage of being inexpensive and easily available. Recently dNLR was carried out as an alternative to NLR to resolve lack of data in multicenter randomized controlled clinical trials. However the external validation showed that its prognostic power is consistently lower than that of NLR or even vanished in some cancers. The reason may be that WBC-neutrophil, mainly consisting of lymphocyte and monocyte which may have inverse effects in terms of predictive value, were put together as the denominator in dNLR. In the present study, we used (WBC-lymphocyte) as the numerator, mainly consisting of neutrophil and monocyte and which are both showed to be associated with poorer prognosis in many cancers. This change augments its prognostic power. As it was demonstrated in our results, the AUC of M/GLR was consistently superior to that of NLR and dNLR in either whole group or subgroups according to TNM stages or different treatments. Also in multivariate analysis the HR for M/GLR was superior to that of NLR and dNLR. Our result suggested that the M/GLR has considerable potential to be adopted universally as a stratification factor in patients with HCC.

The relationships between M/GLR and clinical features are also analyzed in the present study. Spearman’ rank analysis (data not showed) showed that M/GLR was associated with AST (P < 0.001), AFP (P < 0.001), ascites (P < 0.001), tumor number (P = 0.003), diameter lesion (P < 0.001), vascular invasion (P < 0.001) and TNM stage (P < 0.001). These results are almost similar to the previous study^38^ about NLR and dNLR. Patients with higher M/GLR had the tendency to have higher AFP, larger diameter lesions, multiple tumors, and vascular invasion. It suggested that elevated M/GLR might indicate aggressive tumour biology and was associated with poor outcomes after treatment.

The threshold values are varying in different tumor types. The NLR cutoff value in several studies on HCC ranges between 2 and 5^39^,^40^,^41^,^42^. In the present study, we first validated the pre-published cut-off value of 2, determined for the dNLR in the study by Proctor *et al.*^20^, and found a statistically significant association between dNLR ≥ 2 and dNLR < 2 in univariate, but not in multivariate analysis. Therefore, we determined a cut-off value of 1.6 for dNLR to be optimal for the specific cohort of HCC patients. To maintain consistency, we calculated the NLR cut-off value as 2.8 by ROC analysis, which is similar to that previously reported^43^. By using the same method, we first draw a cut-off value of 3.2 for M/GLR. Whether the threshold of M/GLR will be similarly validated in different cancer cohorts remain to be determined. And if it can be adopted in different kinds of cancers, the M/GLR will help the clinician to choose the best individualized treatment.

There are some limitations in the present study. (1) This is a retrospective data collection based on a single institution. (2) Only HCC patients were included in present study, and patient population is based on HBV-related HCC, whether these results can be applied to Western populations wherein HCV, NASH, and other etiologies of liver disease predominate requires further study and comment. (3) The optimal prognostic threshold for the NLR in the range of 2–5 had been consistently validated. It remains to be determined whether a M/GLR of 3.2 will be similarly validated in different cancer cohorts. Therefore, large prospective cohort study with different kinds of cancers should be carried out to verify the prognostic value of M/GLR.

To sum up, we postulated a modified NLR (M/GLR), consisting of leukocyte and lymphocyte, which was superior to NLR and dNLR in prognostic power for patients with HCC, in either whole group or subgroups according to TNM stage or different treatments. Whether M/GLR can be used as an alternative of NLR requires further study and comment.

## Additional Information

**How to cite this article**: Zhou, D. *et al.* A monocyte/granulocyte to lymphocyte ratio predicts survival in patients with hepatocellular carcinoma. *Sci. Rep.* **5**, 15263; doi: 10.1038/srep15263 (2015).

## Supplementary Material

Supplementary Information

## Figures and Tables

**Table 1:** Baseline characteristics and univariate analysis for overall survival in 1061 patients with hepatocellular carcinoma.

**Variables**	**n = 1061**	**Univariate analysis** ***p*****-value**
Age (years)	50(13~80)	0.707
Gender (M/F)	952/109	0.774
WBC (×10^9^/L)	6.2(1.8~24.6)	0.001
Neutrophil count (×10^9^/L)	3.7(0.6~21.5)	<0.001
Lymphocyte count (×10^9^/L)	1.8(0.2~5.8)	<0.001
PLT Count (×10^9^/L)	168(16~553)	0.968
ALT (u/L)	50.2(8~636.4)	<0.001
AST (u/L)	54.4(14~766.5)	<0.001
Albumin (g/L)	40.8(7.6~105.2)	<0.001
Total serum bilirubin (umol/L)	16.2(4.1~222.9)	0.001
ALP (IU/L)	103.6(13~859)	<0.001
AFP (ng/ml)	224.2(0.9~1210000)	<0.001
GGT (U/L)	35.2(13~992)	<0.001
PT (sec)	12.2(9~36.8)	0.027
Ascites (absent/present)	983/78	<0.001
splenomegaly (absent/present)	754/307	0.228
Diameter of largest lesion (cm)	5(1~22.0)	<0.001
Tumor number (solitary/multiple)	657/404	<0.001
Vascular invasion (absent/present)	901/160	<0.001
Child-Pugh grade (A/B)	1019/42	0.005
NLR (≤2.8/>2.8)	788/273	0.001
dNLR (≤1.6/>1.6)	676/385	0.001
M/GLR (≤3.2/>3.2)	764/297	0.001

Abbreviations: WBC = white blood cell count; PLT = platelets; ALT = alanine aminotransferase; AST = aspartate aminotransferase; AFP = alpha-fetoprotein level; ALP = alkaline phosphatase; AFU = Alpha-L-fucosidase; GGT = gamma glutamyl transpeptidase; PT = prothrombin time NLR = neutrophil lymphocyte ratio.

**Table 2:** Multivariate analyses of prognostic factors for overall survival in 1061 patients with hepatocellular carcinoma.

**Variables**	**Multivariate analysis**	
	**Hazard ratio (95% CI)**	***p*****-value**
NLR was used as the covariates		
AFP (ng/ml)	1.49(1.23~1.81)	<0.001
Diameter of largest lesion (cm)	1.89(1.46~2.45)	<0.001
Tumor number (solitary/multiple)	1.68(1.34~2.12)	<0.001
Vascular invasion (absent/present)	1.83(1.44~2.34)	<0.001
NLR (≤2.8/>2.8)	1.32(1.07~1.62)	0.009
dNLR was used as the covariates		
AFP (ng/ml)	1.50(1.24~1.81)	<0.001
Diameter of largest lesion (cm)	1.93(1.49~2.49)	<0.001
Tumor number (solitary/multiple)	1.69(1.34~2.13)	<0.001
Vascular invasion (absent/present)	1.87(1.47~2.39)	<0.001
dNLR (≤1.6/>1.6)	1.26(1.03~1.52)	0.022
M/GLR was used as the covariates		
AFP (ng/ml)	1.50(1.24~1.82)	<0.001
Diameter of largest lesion (cm)	1.86(1.44~2.42)	<0.001
Tumor number (solitary/multiple)	1.72(1.36~2.16)	<0.001
Vascular invasion (absent/present)	1.85(1.45~2.35)	<0.001
M/GLR (≤3.2/>3.2)	1.40(1.14~1.71)	0.001

Abbreviations: AFP = alpha-fetoprotein level; NLR = neutrophil lymphocyte ratio; dNLR = derived nurtrophil lymphocyte ratio; M/GLR = modified nutrophil lymphocyte ratio.
