# Supplementary material for: A monocyte/granulocyte to lymphocyte ratio predicts survival in patients with hepatocellular carcinoma
Source: Sci Rep. 2015 Oct 21;5:15263. doi: 10.1038/srep15263 (PMC4614102; doi:10.1038/srep15263)
Supplement: Supplementary Information [file srep15263-s1.doc]

**TITLE PAGE**

**Title:**  A monocyte/granulocyte to lymphocyte ratio predicts survival in patients with hepatocellular carcinoma.

**Running title:** M/GLR predicts survival for HCC

**Authors:** Dongsheng Zhou M.D.^1, 2 *^, Yaojun Zhang M.D.^1, 2 *^, Li Xu M.D. ^1, 2^, Zhongguo Zhou M.D.^1, 2^, Junting Huang M.D.^1, 2^， Minshan Chen M.D.^1, 2^

**Institutional Affiliation:**

^1^ Department of Hepatobiliary Surgery, Sun Yat-sen University Cancer Center, 651 Dongfeng Road East, Guangzhou 510060, China

^2^ Sun Yat-sen University Cancer Center, State Key Laboratory of Oncology in South China,  Collaborative Innovation Center for Cancer Medicine, Guangzhou 510060, China

^*^ These two authors contribute to this work equally

**Table 1. Comparison of the AUC for outcome prediction among NLR, dNLR, and M/GLR for patients with HCC according to different treatments (Overall treatments, surgery, RFA, and TACE)**

|  |  | AUC | P value | 95%CI |
| --- | --- | --- | --- | --- |
| **Overall Treatments** | | | |  |
| 6 months | | | | |
|  | NLR | 0.589 | 0.021 | 0.510～0.668 |
|  | dNLR | 0.569 | 0.077 | 0.491～0.646 |
|  | M/GLR | 0.595 | 0.014 | 0.516～0.675 |
| 12 months | | | | |
|  | NLR | 0.646 | <0.001 | 0.603～0.689 |
|  | dNLR | 0.625 | <0.001 | 0.581～0.669 |
|  | M/GLR | 0.655 | <0.001 | 0.612～0.697 |
| 24 months | | | | |
|  | NLR | 0.626 | <0.001 | 0.589～0.662 |
|  | dNLR | 0.607 | <0.001 | 0.570～0.644 |
|  | M/GLR | 0.634 | <0.001 | 0.597～0.670 |
| **Surgery** | | | | |
| 6 months | | | | |
|  | NLR | 0.617 | 0.006 | 0.532～0.703 |
|  | dNLR | 0.596 | 0.025 | 0.511～0.681 |
|  | M/GLR | 0.626 | 0.003 | 0.541～0.710 |
| 12 months | | | | |
|  | NLR | 0.614 | <0.001 | 0.552～0.675 |
|  | dNLR | 0.594 | 0.002 | 0.532～0.656 |
|  | M/GLR | 0.623 | <0.001 | 0.562～0.684 |
| 24 months | | | | |
|  | NLR | 0.593 | 0.001 | 0.538～0.647 |
|  | dNLR | 0.571 | 0.008 | 0.517～0.625 |
|  | M/GLR | 0.602 | <0.001 | 0.549～0.656 |
| **Ablation** | | | | |
| 6 months | | | | |
|  | NLR | 0.646 | 0.048 | 0.442～0.851 |
|  | dNLR | 0.638 | 0.09 | 0.408～0.868 |
|  | M/GLR | 0.664 | 0.043 | 0.444～0.883 |
| 12 months | | | | |
|  | NLR | 0.618 | 0.044 | 0.494～0.742 |
|  | dNLR | 0.606 | 0.07 | 0.481～0.732 |
|  | M/GLR | 0.62 | <0.041 | 0.493～0.747 |
| 24 months | | | | |
|  | NLR | 0.602 | 0.027 | 0.512～0.694 |
|  | dNLR | 0.595 | 0.04 | 0.504～0.687 |
|  | M/GLR | 0.606 | 0.021 | 0.514～0.693 |
| **TACE** | | | | |
| 6 months | | | | |
|  | NLR | 0.607 | 0.463 | 0.281～0.933 |
|  | dNLR | 0.561 | 0.676 | 0.251～0.871 |
|  | M/GLR | 0.626 | 0.387 | 0.304～0.948 |
| 12 months | | | | |
|  | NLR | 0.61 | 0.003 | 0.536～0.84 |
|  | dNLR | 0.577 | 0.02 | 0.512～0.662 |
|  | M/GLR | 0.624 | <0.001 | 0.552～0.696 |
| 24 months | | | | |
|  | NLR | 0.569 | 0.05 | 0.5～0.639 |
|  | dNLR | 0.546 | 0.201 | 0.476～0.615 |
|  | M/GLR | 0.581 | 0.024 | 0.511～0.650 |
|  |  |  |  |  |

**Abbreviations**: RFA=radiofrequency ablation; TACE=transcatheter arterial chemoembolization; NLR=neutrophil lymphocyte ratio; dNLR= derived neutrophil lymphocyte ratio; M/GLR= monocyte/granulocyte to lymphocyte ratio.

**Table 2. Comparison of the AUC for outcome prediction among NLR, dNLR, and M/GLR for patients with HCC according to TNM stage** (TNM-I, TNM-II, TNM-III, and TNM-IV)

|  |  | AUC | P value | 95%CI |
| --- | --- | --- | --- | --- |
| **TNM stage I** | | | | |
| 6 months | | | | |
|  | NLR | 0.568 | 0.003 | 0.454～0.681 |
|  | dNLR | 0.565 | 0.076 | 0.446～0.684 |
|  | M/GLR | 0.572 | <0.001 | 0.455～0.689 |
| 12 months | | | | |
|  | NLR | 0.601 | 0.004 | 0.527～0.675 |
|  | dNLR | 0.581 | 0.022 | 0.509～0.654 |
|  | M/GLR | 0.606 | 0.003 | 0.532～0.680 |
| 24 months | | | | |
|  | NLR | 0.602 | 0.001 | 0.542～0.661 |
|  | dNLR | 0.577 | 0.009 | 0.518～0.636 |
|  | M/GLR | 0.61 | <0.001 | 0.550～0.669 |
| **TNM stage II** | | | | |
| 6 months | | | | |
|  | NLR | 0.795 | 0.003 | 0.658～0.933 |
|  | dNLR | 0.784 | 0.004 | 0.654～0.914 |
|  | M/GLR | 0.8 | 0.002 | 0.662～0.937 |
| 12 months | | | | |
|  | NLR | 0.658 | 0.009 | 0.532～0.784 |
|  | dNLR | 0.636 | 0.024 | 0.506～0.765 |
|  | M/GLR | 0.671 | 0.005 | 0.553～0.790 |
| 24 months | | | | |
|  | NLR | 0.585 | 0.071 | 0.491～0.679 |
|  | dNLR | 0.565 | 0.168 | 0.470～0.659 |
|  | M/GLR | 0.595 | 0.044 | 0.502～0.687 |
| **TNM stage III** | | | | |
| 6 months | | | | |
|  | NLR | 0.454 | 0.577 | 0.301～0.606 |
|  | dNLR | 0.426 | 0.367 | 0.275～0.576 |
|  | M/GLR | 0.471 | 0.721 | 0.315～0.626 |
| 12 months | | | | |
|  | NLR | 0.557 | 0.039 | 0.480～0.634 |
|  | dNLR | 0.551 | 0.09 | 0.473～0.629 |
|  | M/GLR | 0.563 | 0.024 | 0.486～0.639 |
| 24 months | | | | |
|  | NLR | 0.578 | 0.003 | 0.525～0.630 |
|  | dNLR | 0.546 | 0.076 | 0.494～0.599 |
|  | M/GLR | 0.591 | <0.001 | 0.539～0.643 |
| **TNM stage IV** | | | | |
| 6 months | | | | |
|  | NLR | 0.552 | 0.609 | 0.313～0.790 |
|  | dNLR | 0.523 | 0.817 | 0.287～0.760 |
|  | M/GLR | 0.574 | 0.465 | 0.348～0.801 |
| 12 months | | | | |
|  | NLR | 0.673 | 0.005 | 0.554～0.792 |
|  | dNLR | 0.634 | 0.029 | 0.513～0.755 |
|  | M/GLR | 0.686 | 0.003 | 0.570～0.801 |
| 24 months | | | | |
|  | NLR | 0.619 | 0.043 | 0.504～0.733 |
|  | dNLR | 0.586 | 0.141 | 0.471～0.701 |
|  | M/GLR | 0.624 | 0.034 | 0.510～0.738 |
|  |  |  |  |  |

**Abbreviations**: RFA=radiofrequency ablation; TACE=transcatheter arterial chemoembolization; NLR=neutrophil lymphocyte ratio; dNLR= derived neutrophil lymphocyte ratio; M/GLR= monocyte/granulocyte to lymphocyte ratio.
